# Ursolic Acid Accelerates Paclitaxel-Induced Cell Death in Esophageal Cancer Cells by Suppressing Akt/FOXM1 Signaling Cascade

**DOI:** 10.3390/ijms222111486

**Published:** 2021-10-25

**Authors:** Ruo Yu Meng, Hua Jin, Thi Van Nguyen, Ok-Hee Chai, Byung-Hyun Park, Soo Mi Kim

**Affiliations:** 1 Department of Physiology, Institute for Medical Sciences, Jeonbuk National University Medical School, Jeonju 54907, Korea; kathymeng1216@gmail.com; 2 School of Pharmaceutical Sciences, Tsinghua University, Beijing 100084, China; jinhuaxy@126.com; 3 Department of Anatomy, Institute for Medical Sciences, Jeonbuk National University Medical School, Jeonju 54907, Korea; thi@jbnu.ac.kr (T.V.N.); okchai1004@jbnu.ac.kr (O.-H.C.); 4 Department of Biochemistry, Jeonbuk National University Medical School, Jeonju 54907, Korea; bhpark@jbnu.ac.kr

**Keywords:** ursolic acid, esophageal squamous cell carcinoma, apoptosis, FOXM1, Akt

## Abstract

Ursolic acid (UA), a pentacyclic triterpenoid extracted from various plants, inhibits cell growth, metastasis, and tumorigenesis in various cancers. Chemotherapy resistance and the side effects of paclitaxel (PTX), a traditional chemotherapy reagent, have limited the curative effect of PTX in esophageal cancer. In this study, we investigate whether UA promotes the anti-tumor effect of PTX and explore the underlying mechanism of their combined effect in esophageal squamous cell carcinoma (ESCC). Combination treatment with UA and PTX inhibited cell proliferation and cell growth more effectively than either treatment alone by inducing more significant apoptosis, as indicated by increased sub-G1 phase distribution and protein levels of cleaved-PARP and cleaved caspase-9. Similar to the cell growth suppressive effect, the combination of UA and PTX significantly inhibited cell migration by targeting uPA, MMP-9, and E-cadherin in ESCC cells. In addition, combination treatment with UA and PTX significantly activated p-GSK-3β and suppressed the activation of Akt and FOXM1 in ESCC cells. Those effects were enhanced by the Akt inhibitor LY2940002 and inverted by the Akt agonist SC79. In an in vivo evaluation of a murine xenograft model of esophageal cancer, combination treatment with UA and PTX suppressed tumor growth significantly better than UA or PTX treatment alone. Thus, UA effectively potentiates the anti-tumor efficacy of PTX by targeting the Akt/FOXM1 cascade since combination treatment shows significantly more anti-tumor potential than PTX alone both in vitro and in vivo. Combination treatment with UA and PTX could be a new strategy for curing esophageal cancer patients.

## 1. Introduction

Esophageal squamous cell carcinoma (ESCC) is a malignant digestive system disease with a high mortality rate. Esophageal cancer is the eighth most common cancer worldwide [1] and the sixth most common cause of cancer-related death due to its poor prognosis [2]. ESCC is difficult to diagnose in the early stage, due to its progression on the onset of initial symptoms, hence the poor prognosis [3,4]. Enzinger et al., reported that the overall five-year survival rate of patients with esophageal cancer is below 15%, and most patients die within the first year after diagnosis [5]. Recently, due to changes in dietary habits and lifestyle, the incidence of esophageal cancer has declined, but the five-year survival rate remains less than 20% [6,7,8]. Resistance to chemotherapy and the toxic effects of traditional chemotherapeutics to normal cells frequently result in treatment failure in patients with esophageal cancer [9,10,11]. Therefore, early diagnostics for esophageal cancer and more accurate, safe, and effective chemotherapy drugs are urgently needed.

Natural products have always been an indispensable and important source in the development of innovative therapeutic drugs for a wide range of diseases, including cancer [12,13,14,15]. Ursolic acid (UA, 3β-hydroxy-12-urs-12-ene-28-oic-acid) is present in various fruits and vegetables and is a crucial part of the human diet [16]. Recently, it has attracted increasing attention due to its comprehensive anticancer properties [17], including cancer cell apoptosis, reduction of cancer cell metastasis, and inhibition of cancer cell proliferation [18,19,20,21,22]. UA can prevent cancer through many signaling pathways, such as ROCK/PTEN, TGF-β1/ZEB1, and MAPK/ERK signaling [19,23,24,25,26,27,28,29,30]. In addition, UA induces cell death with autophagy in patients with esophageal cancer [31]. Therefore, it seems necessary to study various functions of UA in cancer.

Paclitaxel (PTX) was first extracted from Pacific yew in 1971 [32] and later found to induce G2/M cell cycle arrest and apoptosis, accelerating cancer cell death [33]. Since then, PTX has served as an important clinical chemotherapy drug that is widely used to treat various cancers, including breast cancer [34,35], lung cancer [36,37], ovarian cancer [38,39], and esophageal cancer [40,41]. In most cases, combination therapy shows better anti-tumor efficacy than single drug therapy and can reduce chemo-resistance [42,43]. Although PTX shows significant efficacy in esophageal cancer treatment, chemo-resistance reduces its anti-tumor efficacy [44,45]. Therefore, we studied the anti-tumor efficacy of combining UA and PTX in ESCC.

Forkhead box M1 (FOXM1) is a tumorigenic FOX transcription factor [46] believed to have a general role in the development and progression of tumors [47,48,49,50,51] since it regulates the expression of numerous genes with significant roles in cell proliferation, migration, apoptosis, and tumor angiogenesis [52,53]. The overexpression of FOXM1 in tumors of various cancers correlates with late stage, high proliferation rate, and poor prognosis [47,48,49,50,51,54,55,56,57,58,59,60]. In esophageal cancer, FOXM1 has been related to cell growth, proliferation, and metastasis [49,61,62] and plays a key role in both PTX resistance in breast cancer [63] and ovarian cancer [64]. Wang et al. reported that UA inhibits the proliferation of breast cancer cells by suppressing the expression of FOXM1 [65]. Therefore, it can be hypothesized that UA can increase sensitivity to cancer cells that are resistant to PTX. FOXM1 is an important downstream gene of the Akt signaling pathway in gastric cancer [66] and can induce cell death by regulating Akt signaling [67,68,69,70]. However, the underlying molecular mechanisms by which UA regulates the Akt/FOXM1 signaling pathway and enhances the anti-tumor efficacy of PTX in ESCC are not fully understood. In this study, we demonstrate that UA inhibits esophageal cancer cell growth, cell proliferation, and metastasis; induces apoptosis via the Akt/FOXM1 pathway; and potentiates the anti-tumor efficacy of PTX both in vivo and in vitro.

## 2. Results

### 2.1. Inhibition of Cell Proliferation by UA and PTX

**Figure 1:**
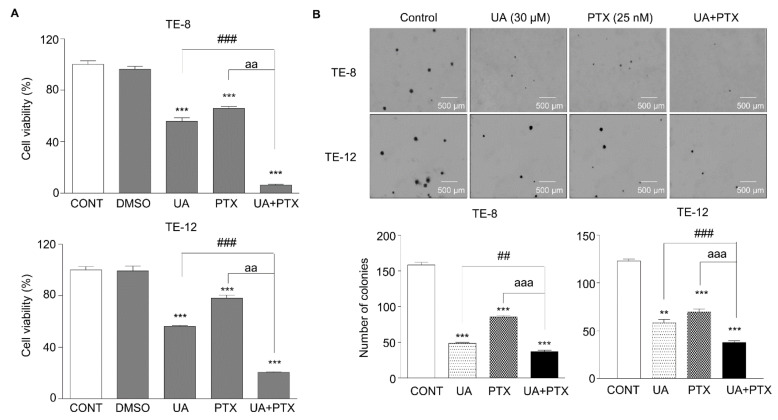
UA and PTX inhibited cell proliferation in ESCC cells. TE-8 and TE-12 cells were treated with UA 30 μM, PTX 25 nM, or both. (**A**). Cell viability was assessed using the WST-1 assay. (**B**). A colony formation assay was performed to study cell proliferation. Images were taken by microscopy, and the colonies were counted in more than five areas per well. Data are the mean (SE) of >three independent experiments with triplicate dishes. *, compared with the control group; ^#^, UA compared with the UA plus PTX combination group; ^a^, PTX group compared with the UA plus PTX combination group. ^##^, ^aa^, ** *p* < 0.01 and ^###^, ^aaa^, *** *p* < 0.001. Scale bar: 500 µm. CONT, control; DMSO, dimethyl sulfoxide; UA, ursolic acid 30 μM; PTX, paclitaxel 25 nM; UA+PTX, combination treatment of ursolic acid 30 μM and paclitaxel 25 nM.

Cell viability was determined using the WST-1 assay. To assess the combined effects of UA and PTX on cell proliferation, TE-8 and TE-12 cancer cells were treated with UA (0, 10, 20, 30, 40, 50 μM) and PTX (0, 10, 20, 30, 40, 50 nM) to calculate the IC50 values of each drug. The IC50 of UA was 31.66 ± 0.52 and 34.48 ± 0.75 in TE-8 and TE-12 cell lines, and that of PTX was 28.58 ± 1.25 and 31.09 ± 1.15, respectively. Based on these data, UA was selected as 30 μM and PTX as 25 nM. Thus, TE-8 and TE-12 cancer cells were treated with UA 30 μM, PTX 25 nM, or a combination of both. The combination treatment resulted in 80–90% inhibition of cell growth, which was higher than in the control group (Figure 1A). Both the size and number of colonies in the UA and PTX combination groups decreased significantly (Figure 1B). These results suggest that this drug combination produces a considerably greater inhibitory effect on esophageal cell proliferation than treatment with either UA or PTX alone.

### 2.2. Induction of Apoptosis by UA and PTX

**Figure 2:**
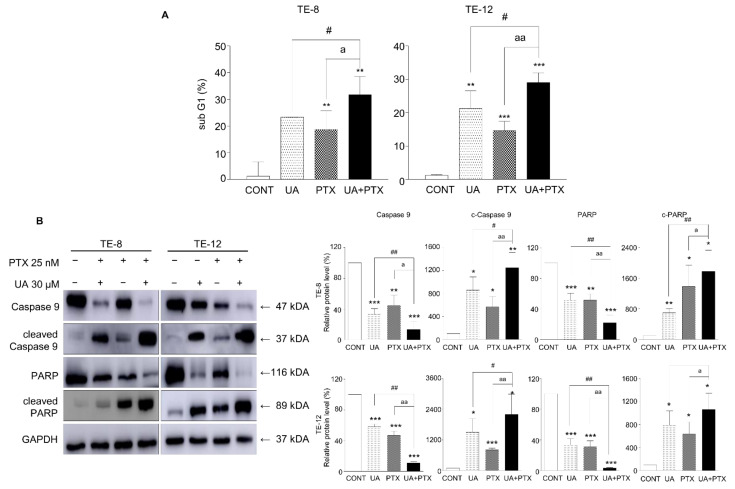
UA and PTX induced apoptosis in ESCC cells. (**A**). The sub-G1 phase population was detected using a FACS analysis. (**B**). The expression of cleaved-PARP, cleaved caspase 9, PARP, and caspase 9 was measured by western blotting after treatment with UA 30 μM, PTX 25 nM, or both. GAPDH is the internal control. Quantification of bands was calculated using ImageJ. Data are expressed as the mean ± SE. *, compared with the control group; ^#^, UA compared with the UA plus PTX combination group; ^a^, PTX group compared with the UA plus PTX combination group. ^#^, ^a^, * *p* < 0.05; ^##^, ^aa^, ** *p* < 0.01 and *** *p* < 0.001. CONT, control; UA, ursolic acid 30 μM; PTX, paclitaxel 25 nM; UA+PTX, combination treatment of ursolic acid 30 μM and paclitaxel 25 nM.

To test the apoptotic effects of UA and PTX on TE-8 and TE-12 cells, FACS analysis was performed to examine the sub-G1-phase population. The sub-G1-phase population accumulated significantly more in the group treated with the combination of UA and PTX than in the groups treated with single agents (Figure 2A). We next detected the protein expression of apoptotic proteins: cleaved-PARP, PARP, cleaved caspase-9, and caspase-9. Treatment with UA 30 μM or PTX 25 nM alone enhanced cleaved-PARP and cleaved caspase-9 protein expression compared with the control cells and decreased the expression of the caspase-9 and PARP proteins (Figure 2B). Combining UA and PTX markedly increased cleaved-PARP and cleaved caspase-9 expression and markedly decreased caspase-9 and PARP expression. These results indicate that UA improved the PTX induced apoptosis of esophageal cancer cells.

### 2.3. Cell Cycle Arrest Was Induced by UA and PTX in ESCC

**Figure 3:**
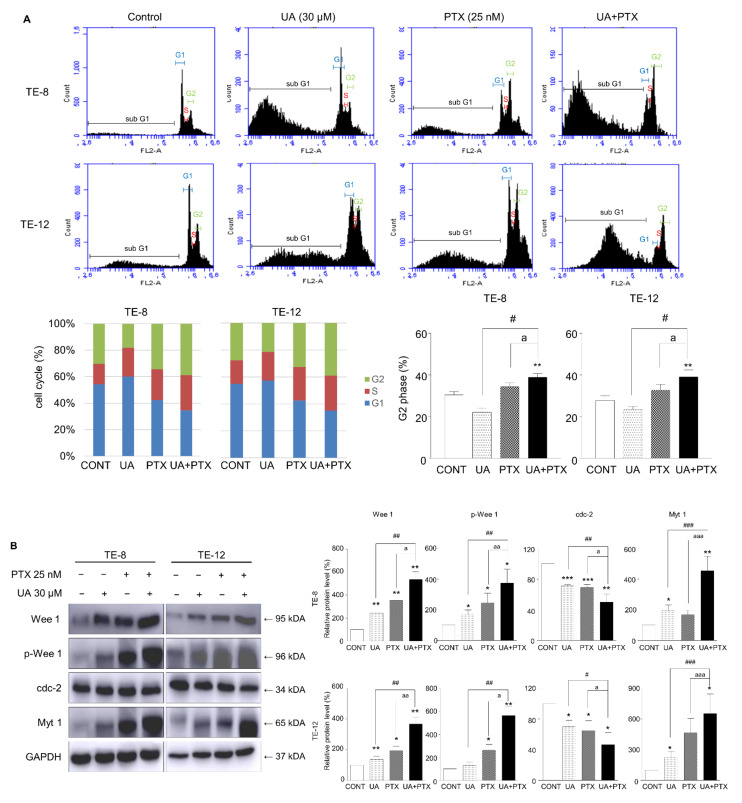
UA and PTX induced G2-phase cell cycle arrest in ESCC cells. (**A**). The cell cycle analysis was conducted using FACS. The combination treatment induced more significant G2-phase arrest than the single treatments. (**B**). Expression of p-Wee1, cdc2, and Myt1 was detected by western blotting. GAPDH is the internal control. Data are expressed as the mean ± SE. Quantification of bands was calculated using Image J. *, compared with the control group; ^#^, UA compared with the UA plus PTX combination group; ^a^, PTX group compared with the UA plus PTX combination group. ^#^, ^a^, * *p* < 0.05; ^##^, ^aa^, ** *p* < 0.01 and ^###^, ^aaa^, *** *p* < 0.001. CONT, control; UA, ursolic acid 30 μM; PTX, paclitaxel 25 nM; UA+PTX, combination treatment of ursolic acid 30 μM and paclitaxel 25 nM.

To investigate how combining UA and PTX affected cell cycle progression, we analyzed the FACS cell cycle in TE-8 and TE-12 cells. The cell cycle distribution was estimated 48 h after treatment with UA 30 μM, PTX 25 nM, or both. Combining UA and PTX induced a significant increase in the G2-phase population (Figure 3A). A western blot analysis showed the effects of cell cycle-related proteins following treatment with UA and PTX. Treatment with a single-agent increased the Myt 1, Wee1, and p-Wee1 protein levels and decreased the cdc-2 protein level, while combination treatment with UA and PTX produced more significant alterations in the cdc-2, Myt 1, Wee1, and p-Wee1 protein levels (Figure 3B). These results indicate that combining UA and PTX induces noticeable G2 phase arrest in esophageal cancer cells.

### 2.4. Inhibition of Invasion and Metastasis in ESCC by UA and PTX

**Figure 4:**
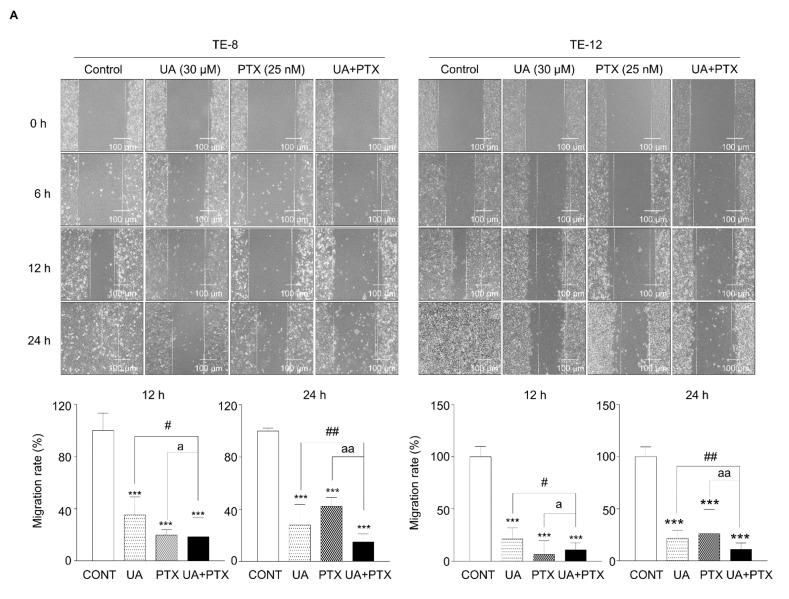

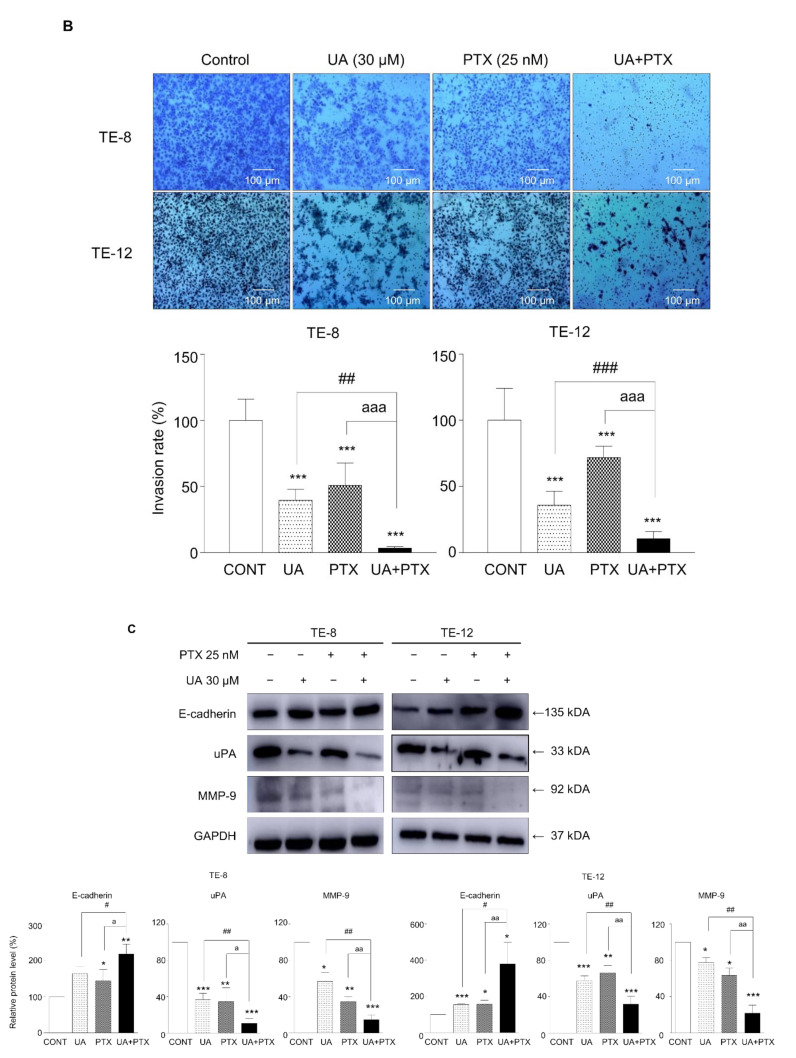
UA and PTX inhibited cell invasion and metastasis in ESCC cells. (**A**) Wound healing assay was designed to assess cell migration. Images were taken at 12 h and 24 h. (**B**) Matrigel-migration assay was performed to detect the invasion rate. The combination treatment inhibited the invasion rates significantly better than the single agents. (**C**). Effects of UA and PTX on migration-related proteins. Expression of E-cadherin, uPA and MMP-9 was examined by western blotting. GAPDH is the internal control. Quantification of bands was calculated using ImageJ. *, compared with the control group; ^#^, UA compared with the UA plus PTX combination group; ^a^, PTX group compared with the UA plus PTX combination group. ^#^, ^a^, * *p* < 0.05; ^##^, ^aa^, ** *p* < 0.01 and ^###^, ^aaa^, *** *p* < 0.001. Scale bar: 100 µm. CONT, control; UA, ursolic acid 30 μM; PTX, paclitaxel 25 nM; UA+PTX, combination treatment of ursolic acid 30 μM and paclitaxel 25 nM.

To research how UA and PTX affect esophageal cancer cell migration and invasion, a wound healing assay and Matrigel invasion assay were performed. The migration ability of esophageal cancer cells was markedly reduced by treatment with UA and PTX together, compared with single-agent treatment (Figure 4A). Treatment with UA or PTX alone reduced the invasion function, and the combination treatment had a markedly greater anti-invasive effect at 12 h and 24 h (Figure 4B). In addition, western blotting was used to explore metastasis-related protein expression. The E-cadherin protein level increased more noticeably with the combination treatment than with the single-agent treatment, whereas the uPA and MMP-9 protein levels decreased in the combination group (Figure 4C). Thus, UA potentiates the inhibitory effect of PTX on cell invasion and metastasis in esophageal cancer cells by targeting uPA, MMP-9, and E-cadherin.

### 2.5. Downregulation of Akt Signaling Pathway by UA and PTX

**Figure 5:**
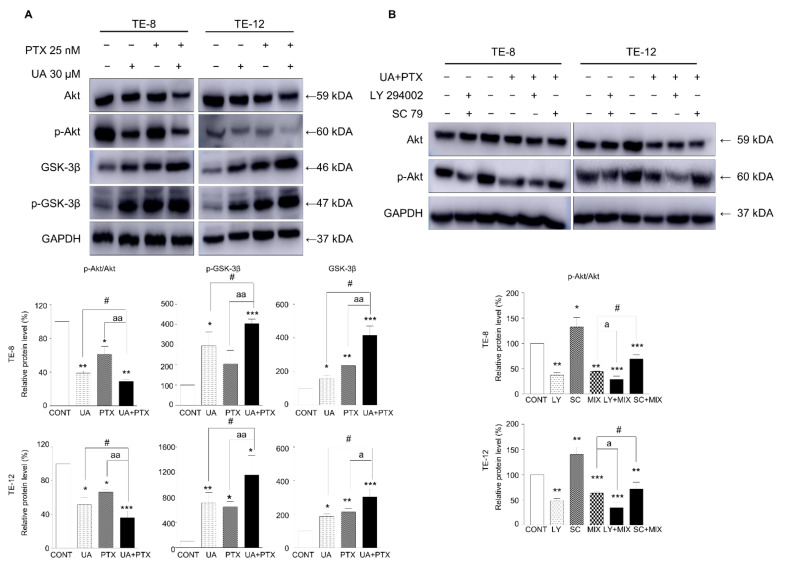
UA and PTX downregulated Akt signaling in ESCC cells. (**A**). The expression of Akt, p-Akt, GSK-3β, and p-GSK-3β was examined by western blotting in the presence of UA 30 μM, PTX 25 nM, or both. (**B**). In the presence of an Akt inhibitor (LY294002) or Akt agonist (SC79), Akt and p-Akt were measured by western blotting with or without the UA and PTX combination treatment. The cells were pretreated with LY294002 (10 μM) or SC79 (10 μM) for 2 h and then treated with UA 30 μM and PTX 25 nM for 48 h. GAPDH is the internal control. Data are expressed as the mean ± SE. Quantification of bands was calculated using Image J. *, compared with the control group; ^#^, UA compared with the UA plus PTX combination group; ^a^, PTX group compared with the UA plus PTX combination group. ^#^, ^a^, * *p* < 0.05; ^aa^, ** *p* < 0.01 and *** *p* < 0.001. CONT, control; LY, LY294002; SC, SC79; MIX, combination treatment of ursolic acid 30 μM and paclitaxel 25 nM; UA, ursolic acid 30 μM; PTX, paclitaxel 25 nM.

To test whether the Akt signaling pathway was altered by the UA and PTX combination treatment in esophageal cancer cells, p-Akt (ser473), Akt, GSK, and GSK-3β protein levels were examined using western blotting. Treatment with UA or PTX alone markedly decreased the p-Akt and Akt protein levels compared with the control group, and combining the two treatments produced a greater reduction in the Akt and p-Akt proteins (Figure 5A). Additionally, treatment with UA or PTX enhanced the protein levels of GSK and p-GSK-3β, and combining the treatments induced markedly greater expression of GSK and p-GSK-3β protein in esophageal cancer cells. To further test whether those results occurred via Akt signaling, Akt/p-Akt protein levels were measured following treatment with an Akt inhibitor (LY294002) and Akt activator (SC79). The p-Akt protein level was significantly accelerated by the Akt inhibitor and inverted by the Akt activator in ESCC cells (Figure 5B). Thus, UA enhanced the inhibition effect of PTX on cell proliferation and metastasis in esophageal cancer cells by mediating the Akt signaling pathway.

### 2.6. Downregulation of FOXM1 Levels by UA and PTX

**Figure 6:**
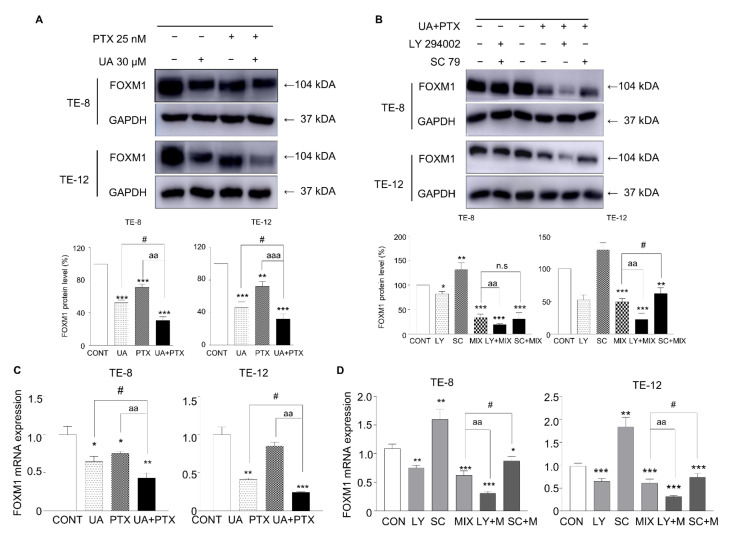
UA and PTX inhibited FOXM1 expression in ESCC cells. (**A**,**B**) Protein expression of FOXM1 was detected by western blotting. (**C**,**D**) mRNA levels of FOXM1 were measured by real-time PCR and analyzed by the ΔΔct method. GAPDH is the internal control. Quantification of bands was calculated using Image J. Data are expressed as the mean ± SE. *, compared with the control group; ^#^, UA compared with the UA plus PTX combination group; ^a^, PTX group compared with the UA plus PTX combination group. ^#^, * *p* < 0.05; ^aa^, ** *p* < 0.01 and ^aaa^, *** *p* < 0.001. CONT, control; LY, LY294002; SC, SC79; MIX, combination treatment of ursolic acid 30 μM and paclitaxel 25 nM; UA, ursolic acid 30 μM; PTX, paclitaxel 25 nM.

FOXM1 is a key transcription factor regulating cancer cell growth and metastasis. To investigate whether UA and PTX affect the functioning of genes downstream of the Akt signaling pathway, we explored the expression of the downstream FOXM1 gene in TE-8 and TE-12 cells. The protein and mRNA levels of FOXM1 decreased significantly after treatment with UA or PTX (Figure 6A,B). The combination group showed a more effective reduction in FOXM1 expression than either single treatment group. To further test whether those results occurred via Akt signaling, FOXM1 protein levels were measured following treatment with the Akt inhibitor LY294002 and the Akt activator SC79. The protein level of FOXM1 was significantly accelerated by the Akt inhibitor and inverted by the Akt activator in ESCC cells (Figure 6C). These findings suggest that the UA and PTX combination we tested induced more noticeable inhibition of FOXM1, mediated through the Akt pathway, than either single-agent treatment.

### 2.7. Treatment with UA Plus PTX Inhibited Tumor Growth in Xenograft Animal Model

After confirming that UA enhances the anticancer effect of PTX in esophageal cancer cells in vitro, we decided to test whether that efficacy could be verified in a murine xenograft model in vivo.

**Figure 7:**
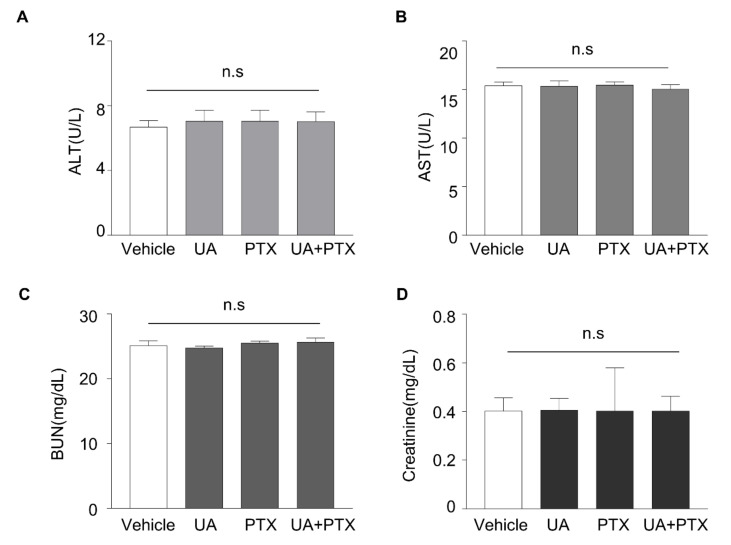
Toxic effect of UA and PTX in xenograft animal model. Xenograft model was performed with the TE-8 cell line. After tumors were established, the mice were injected with DPBS, UA, PTX, or UA plus PTX for three weeks. The serum biochemical indexes for ALT (**A**), AST (**B**), BUN (**C**), and creatinine (**D**) were used for the drug safety evaluation. n.s, not significant; Vehicle, DPBS; UA, ursolic acid 30 μM; PTX, paclitaxel 25 nM; UA+PTX, combination treatment of ursolic acid 30 μM and paclitaxel 25 nM.

**Figure 8:**
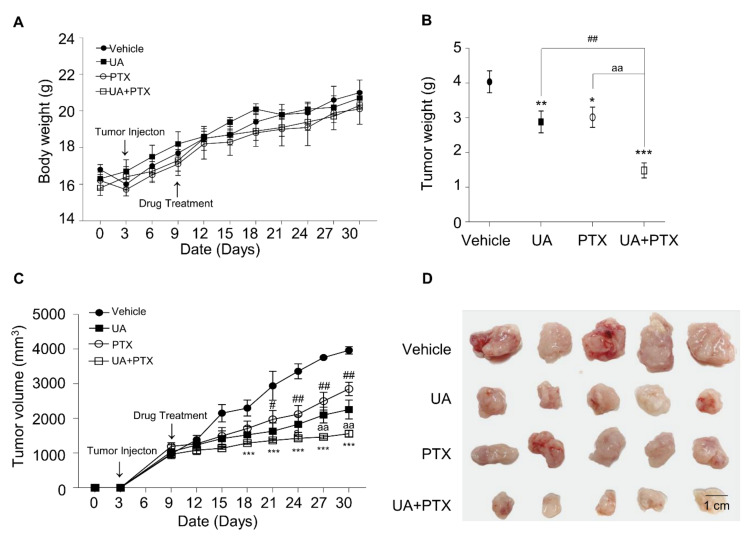
UA and PTX inhibited tumor growth in TE-8 xenografts *in vivo*. Body weight (**A**) and tumor volume (**C**) were measured every three days. Tumor weight (**B**) and tumor size (**D**) were measured after euthanasia. ^#^, UA-treatment group compared with the UA plus PTX combination treatment group; ^a^, PTX treatment group compared with the UA plus PTX combination treatment group; *, compared with the vehicle group. ^#^, * *p* < 0.05; ^##^, ^aa^, ** *p* < 0.01 and *** *p* < 0.001 compared with the vehicle. Vehicle, DPBS; UA, ursolic acid 30 μM; PTX, paclitaxel 25 nM; UA+PTX, combination treatment of ursolic acid 30 μM and paclitaxel 25 nM.

**Figure 9:**
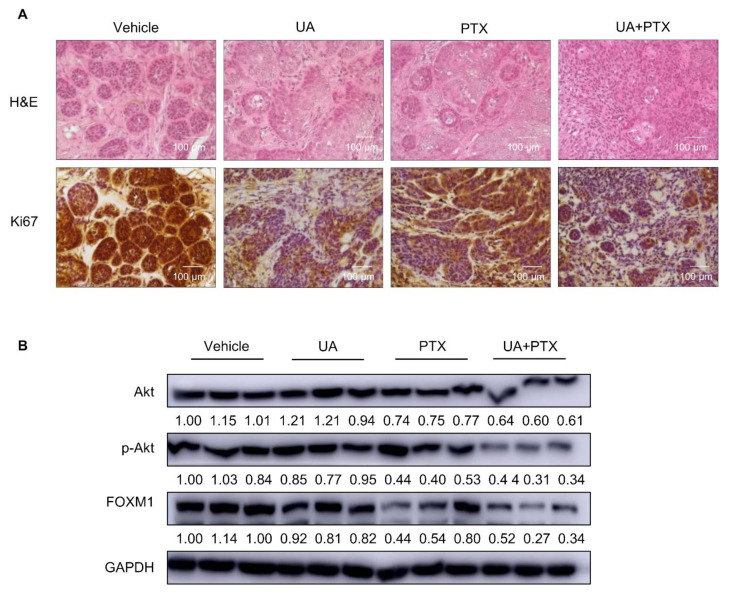
Histological examination of tumor tissue (**A**). Tumor tissues were examined using hematoxylin and eosin staining (upper row) and Ki-67 immunohistochemistry staining (lower row). Scale bar: 100 μm. (**B**). Expression of Akt/p-Akt/FOXM1 in tumor tissue was examined by western blotting. GAPDH is the internal control. Quantification of bands was calculated using Image J. Vehicle, DPBS; UA, ursolic acid 30 μM; PTX, paclitaxel 25 nM; UA + PTX, combination treatment of ursolic acid 30 μM and paclitaxel 25 nM.

After TE-8 tumor cells were injected into xenograft mice, the tumors were allowed to grow until they were 200 mm^3^, and then the mice were divided into four groups: UA, PTX, a combination of UA and PTX, and control. To evaluate the safety of UA and PTX, we measured biochemical markers for ALT, AST, BUN, and creatinine levels in the mouse serum. As shown in Figure 7, the ALT, AST, BUN, and creatinine levels did not differ meaningfully between the control group and the drug treatment groups. As shown in Figure 8A, body weight did not differ significantly between the control group and the drug treatment groups. Compared with UA or PTX treatment alone, the combination treatment significantly inhibited tumor weight (Figure 8B), tumor volume (Figure 8C), and tumor size (Figure 8D) in the xenograft model animals. As shown in Figure 9A, hematoxylin and eosin (H&E) staining data indicated that tumor clusters were present distant from the main mass, and in the control group, they usually became well differentiated. However, poorly differentiated carcinoma composed of rounded cell clusters without clear-cut squamous differentiation was present in the combination treatment group. In addition, our immunohistochemistry data show that Ki-67 positive cells were highly expressed in tumor clusters in the control group, whereas they were poorly expressed in the UA plus PTX treatment group. To further test whether the Akt/p-Akt and FOXM1 protein levels changed in the xenograft animal model, we measured those levels in the tumor tissue (Figure 9B). The experimental results were similar to those from the in vitro experiments. Compared with the single-agent treatment groups, the combination treatment group showed a more notable reduction in Akt/p-Akt and FOXMI protein expression, suggesting that combination therapy with UA and PTX significantly inhibits tumor growth in xenograft animal experiments and has anti-tumor potential in ESCC.

## 3. Discussion

ESCC is the eighth most common cancer worldwide [1,2]. Limitations of traditional chemotherapy such as drug resistance and toxicity lead to frequent treatment failure in esophageal cancer [9,10,11]. Therefore, more accurate diagnostics and new chemotherapy regimens are needed for esophageal cancer treatment.

Chemotherapy is an essential therapeutic option for cancer patients at all stages [71,72]. PTX is one of the most widely used cytotoxic agents for cancer treatment, including esophageal cancer. It targets tubulin and inhibits microtubule assembly, chromosome segregation, and cell division to stop cancer cell growth [73,74,75]. Although PTX is extensively used to treat many cancers, it still has severe side effects and many restrictions [40,44,75]. The concomitant application of different chemotherapeutics is a major treatment strategy for cancer patients. Research on the potential of natural compounds as novel chemo-adjuvants is being actively conducted [15,76,77]. UA, a pentacyclic triterpenoid, can be extracted from several plants and fruits [16]. The cytotoxic potential of UA has been demonstrated both in vitro and in vivo [17,20,22]. Numerous studies have reported that UA regulates the apoptosis, proliferation, metastasis, and cell cycle of different cancer cells through several signaling pathways, including Akt/PI3K [28,29], NF-Κβ [27], and STAT3 [26,78]. In agreement with those studies, our previous study showed that UA effectively inhibited cell viability and induced ROS-mediated autophagy in esophageal cancer cells through the Akt/mTOR signaling pathway [31], suggesting that UA might act as a potential anti-tumor reagent in esophageal cancer. Although PTX shows significant efficacy as a common esophageal cancer chemotherapy drug, chemo-resistance reduces its effects [44,45]. However, Zhang et al., showed that UA enhanced the therapeutic effects of oxaliplatin in colorectal cancer by ROS-mediated inhibition of drug resistance [79]. Therefore, a combination therapy might offer better anti-tumor efficacy than single drug therapy and reduce chemo-resistance [42,43].

In this study, we researched the anti-tumor efficacy of combining UA and PTX and the underlying molecular mechanisms of that combination in esophageal cancer cells. This study is the first to demonstrate the synergistic anticancer effects of combining UA with PTX in esophageal cancer cells. We determined that their combination conferred much greater inhibition of cell proliferation in TE-8 and TE-12 cells than either treatment alone. The sub-G1 phase detection and apoptosis-related protein expression data show that UA induced apoptosis and that the combination of UA and PTX accelerated that apoptotic effect in esophageal cancer cells. In addition, the combination of UA and PTX displayed G2 cell cycle arrest, even though UA alone induced G1 cycle arrest. Moreover, the wound healing assay and Matrigel invasion assay clarified that the combination of UA and PTX targeted uPA, MMP-9, and E-cadherin and thereby produced more significant migration inhibitory effects than the single treatments. These observations are consistent with those of a previous study that combined UA with PTX in human gastric cancer cells and found a synergistic effect via the induction of apoptosis by suppressing cyclooxygenase-2 [80]. Our study suggests that UA enhances the cytotoxic effect of PTX in ESCC cells, as well as in gastric cancer cells. Our in vivo evaluation in a murine TE-8 xenograft model of esophageal cancer also showed that a combination of UA and PTX suppressed tumorigenesis more effectively than UA or PTX alone. Furthermore, a biochemical analysis did not indicate liver or kidney impairment in any of the treatment groups. This result supports the notion that the dose of UA we tested causes no systemic toxicity in mice, suggesting that UA could suppress tumor growth without any side effects. Moreover, a histological analysis showed notable changes in the tumors. In H&E staining, poorly differentiated carcinomas composed of rounded cell clusters were observed in the control group, and treatment with a combination of UA and PTX markedly increased the quantity of apoptotic and necrotic cells compared with UA or PTX alone. A similar pattern was found in the immunohistochemistry data. The control group expressed a tremendous number of Ki-67 positive cells, and significantly fewer were expressed in the group treated with the UA and PTX combination. Taken together, these results suggest that combining UA and PTX treatment produces synergistic inhibition of proliferation and metastasis in vivo and in vitro, demonstrating the chemo-sensitizing effect of UA in esophageal cancer.

FOXM1 is a FOX transcription factor that regulates multiple processes in cancer [46,51]. FOXM1 is overexpressed in various cancer types and positively correlates with poor prognosis [47,48,49,50,51,54,55,56,57,58,59,60]. FOXM1 expression has evident correlations with esophageal cancer pathology [49,62]. Specifically, FOXM1 functions as a downstream gene in mTOR/Akt signaling and plays an important role in cell survival and metastasis [66,68]. In addition, FOXM1 has been shown to play a key role in PTX resistance in breast cancer and ovarian cancer [63,64]. Wang et al. demonstrated that UA induces apoptosis by inhibiting expression of FOXM1 in human breast cancer cells [65]. Yan et al. also reported that activation of Akt/FXOM1 signaling present sorafenib resistance to liver cancer cells [81], suggesting that Akt/FOXM1 plays a very important role in drug resistance in various cancers. In fact, FOXM1 was found to be significantly increased in cancerous tissue samples of patients with ESCC [62]. Therefore, targeting Akt/FOXM1 is a potential way to cure esophageal cancer, we further examined whether UA and PTX regulate the Akt/FOXM1 signaling pathway in ESCC cells. UA reduced Akt and the phosphorylation of Akt expression and increased the GSK and the phosphorylation of GSK-3β in ESCC cells. Moreover, the combination of UA and PTX markedly increased the suppression of Akt function and the induction of GSK and GSK-3β phosphorylation in ESCC cells. The protein level of p-Akt in ESCC cells decreased upon treatment with the UA and PTX combination, and that decrease was significantly suppressed by the Akt inhibitor LY294002 and induced by the Akt activator SC79. These findings are consistent with those of other studies, which demonstrated that UA’s anticancer activity involves downregulating the phosphorylation of Akt in oral, lung, ovarian, breast, prostate, and bladder cancer cells [18,20,21,27,28,82,83]. Since FOXM1 is a downstream signal of the Akt pathway and that FOXO3a suppresses FXOM1 expression when Akt signaling is inactivated [70,84], we further measured FOXM1 protein and mRNA levels. The combination of UA and PTX inhibited *FOXM1* mRNA and protein expression in ESCC cells considerably more than either single treatment. The reduced FOXM1 levels were further decreased significantly by LY294002 and increased by SC79. In our in vivo experiments, the combination of UA and PTX significantly inhibited the expression of p-Akt and FOXM1 proteins compared with the single treatments in TE-12 xenograft tumors. These observations indicate the anticancer effect of UA in an in vivo experiment, suggesting that UA might act as a potential anti-tumor reagent in esophageal cancer cells. The downregulation of Akt by the combination of UA and PTX promotes the suppression of FOXM1 expression, which inhibits growth and metastasis and induces apoptosis in ESCC cells.

**Figure 10:**
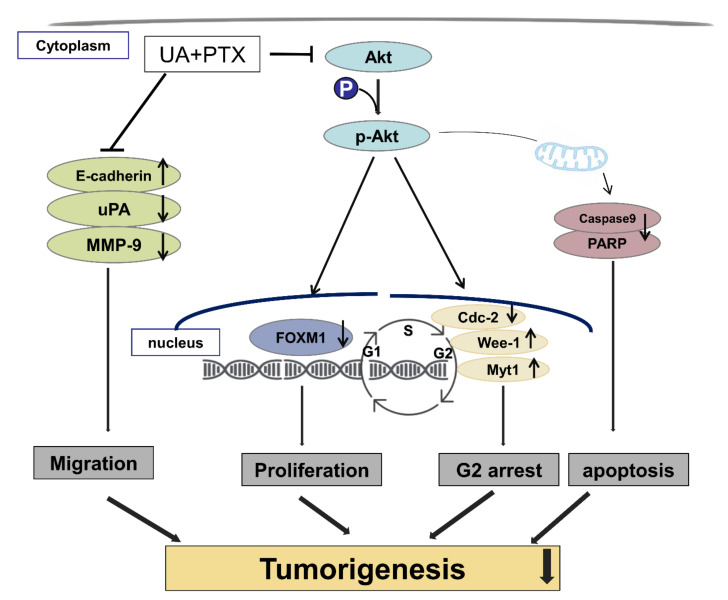
Schematic diagram represents that UA enhances PTX induced cell death in ESCC cells by inactivating Akt/Foxm1 signaling cascade.

In summary, we have demonstrated how a new UA and PTX combination treatment suppresses esophageal cancer cell growth and tumorigenesis by inhibiting FOXM1 expression via the Akt signaling pathway (Figure 10). However, UA dose not seems to bind directly to any proteins (Akt, GSK, FOXM1 etc.) to potentiate their activity in ESCC cells. UA may mediate the enhancement of PTX induced inhibition of cancer cell growth through indirect mechanisms through inactivation of Akt/FOXM1 signaling pathway. Therefore, combination treatment with UA and PTX could be a new strategy for curing esophageal cancer patients.

## 4. Materials and Methods

### 4.1. Cell Culture and Experimental Reagents

The TE-8 and TE-12 esophageal cancer cell lines were purchased from the Korean Cell Line Bank (Seoul National University, Seoul, Korea). The TE-8 and TE-12 cell lines were cultured in RPMI-1640 medium (Gibco, Grand Island, NY, USA) with 10% fetal bovine serum (FBS, WELGENE, Gyeongsan-si, Korea) and 1% penicillin–streptomycin (SIGMA, St. Louis, MO, USA) under standard conditions at 37 °C in a 5% CO_2_ humidified atmosphere. Primary antibodies for caspase-9, cleaved caspase 9, PARP, cleaved-PARP, phosphorylated-Akt, GSK-3β (#9315), phosphorylated-GSK-3β, cdc-2, Myt1, Wee1 (#4936), p-Wee1, MMP-9 (#3852) and GAPDH were obtained from Cell Signaling Technology (Denver, MA, USA), and antibodies against Akt, uPA, FOXM1, and E-cadherin were obtained from Santa Cruz Biotechnology Inc. (Dallas, CA, USA). UA was purchased from Cayman Chemical Company (Ann Arbor, MI, USA). PTX and LY294002 were bought from Sigma Chemical Company (St. Louis, MO, USA). SC79 was purchased from Tocris Bioscience (Bristol, UK).

### 4.2. WST-1 Assay

TE-8 and TE-12 cells were seeded in 96-well plates in RPMI-1640 medium without FBS and allowed to attach to the wells. The next day, the cells were treated with UA 30 μM, PTX 25 nM, or UA plus PTX combination was administered for 48 h. Cell viability was measured using an EZ-CYTOX assay kit (EZ-Cytox, DOGEN, Seoul, South Korea). The following processes were performed according to the manufacturer’s instructions. We added 10 µL of EZ-CYTOX and 100 µL of RPMI-1640 medium (Gibco, Grand Island, NY, USA) to each well and shook them gently for 5 min in the dark. Next, we incubated them for 2 h at 37 °C in the dark and then detected the absorbance at 450 nM using an Epoch microplate reader (Bio Tek, Winooski, VT, USA). These experiments were repeated at least three times.

### 4.3. Soft Agar Colony Formation Assay

A bottom layer of soft agar (1%) was added to a six-well plate and allowed to solidify at room temperature. The top layer (0.7%) was mixed with 10^5^ cells/well and gently added to the bottom gel in a single cell suspension. The cells were divided into the necessary groups and covered with medium containing UA 30 μM, PTX 25 nM, or both. The cells were cultured in an incubator at 37 °C with 5% CO_2_ for four weeks, with the medium changed twice each week. Images of colonies were taken by microscopy. Colonies of ≥30 cells were counted, and the number of colonies was counted in more than five areas per well. All experiments were performed three times in three independent replicates.

### 4.4. Western Blot Analysis

TE-8 and TE-12 cells were treated with UA 30 μM, PTC 25 nM, or both for 48 h. Then the cells were collected and re-suspended in RIPA lysis buffer (Thermo Fisher Scientific Inc., Waltham, MA, USA) in a protease inhibitor cocktail and phosphatase inhibitor cocktail (Pierce, Rockford, IL, USA). The cell pellets were put on ice for 30 min and then centrifuged at 13,200× *g* for 20 min at 4 °C. The supernatant was collected after centrifugation, and the protein concentration was quantified using a BSA protein assay kit (Pierce Biotechnology, Inc., Rockford, IL, USA). Protein samples were separated in SDS-PAGE gels and transferred to PVDF membranes (GE Healthcare Life Sciences, Buckinghamshire, UK). The membranes were incubated with specific primary antibodies overnight at 4 °C and then covered with peroxidase-conjugated secondary antibodies for 2 h at 4 °C. Images were developed by using Chemiluminescent HRP Substrate (Millipore Corporation, Billerica, MA, USA). The image bands were quantified using ImageJ software (1.53K14). The following antibodies were used: cleaved-PARP, PARP, cleaved caspase 9, caspase 9, Akt, p-Akt, p-GSK-3β, FOXM1, uPA, E-cadherin, cdc-2, Myt1, p-Wee1, and GAPDH.

### 4.5. Wound Healing Assay

TE-8 and TE-12 cells were seeded in six-well plates and allowed to grow to 80% confluence. The cells were treated with UA 30 μM with or without PTX 25 nM. Wounds were scratched in each monolayer with a pipette tip. The wounds were monitored and images were taken at different time points (0, 6, 12, 24 h) in the same position under light microscopy. The length of wounds was random measured in five area each images by image J software and then calculated the migration rate and made a graph. These experiments were repeated at least three times.

### 4.6. Transwell Matrigel Invasion Assay

BD BioCoat^TM^ Matrigel^TM^ Invasion Chambers (BD Biosciences, San Jose, CA, USA) were used for the in vitro cell invasion assay. We rehydrated the Matrigel-coated chambers with medium at 37 °C in a 5% CO_2_ atmosphere for 2 h. Then, TE-8 and TE-12 cells were seeded in the chambers at 2.5 × 10^4^/well in 500 μL of RPMI-1640 medium with 1% FBS. Medium with 10% FBS with and without the drugs was then placed in the wells. After 48 h of incubation, the chambers were washed with DPBS and stained with a Diff-Quik kit (Sysmex Corp., Kobe, Japan). Images were taken, and cells in the lower side of the chamber membrane were counted by microscope in five randomly selected fields. The invasion rates were then calculated as previously described. The experiments were repeated at least three times.

### 4.7. RNA Isolation and Real-Time PCR

Total RNA was extracted from TE-8 and TE-12 cells after drug or medium treatment. cDNA reverse transcription was executed with a PrimeScript^TM^ RT reagent kit (Takara Bio Inc., Otsu, Shiga, Japan) according to the manufacturer’s instruction. Quantitative real-time PCR was performed using SYBR Premix Ex Taq (Takara Bio Inc., Otsu, Shiga, Japan) in an ABI Prism 7900 Sequence Detection System (Applied Biosystems, Foster City, CA, USA). The PCR program was initiated at 95 °C for 30 s, followed by 40 cycles of 95 °C for 15 s and 60 °C for 1 min. The results were calculated from threshold cycle numbers using the ΔΔct method. The primer sequences were as follows: FOXM1 sense, 5′ACGTCCCCAAGCCAGGCTC3’ and antisense, 5′CTACTGTAGCTCAGGAATAA3′; *GAPDH* sense, 5′GTCTCCTC TGACTTCAACAGCG3′ and antisense, 5′ACCACCCTGTT GCTGTAGCCAA3′.

### 4.8. Cell Cycle Analysis

To determine the effect of UA and PTX on cell cycle distribution, the assay was performed using the cell cycle kit (Millipore, Burlington, MA, USA cat # MCH100106). TE-8 and TE-12 cells were collected in DPBS after treatment with UA 30 μM, with or without PTX 25 nM. The cells were fixed using 75% ethanol for 2 h at −20 °C. Then we removed the ethanol and rinsed the cells with DPBS. Next, the cells were incubated with RNase A for 15 min at 37 °C and then stained with propidium iodide (Sigma Chemicals, St. Louis, MO, USA) for 30 min at room temperature in the dark. After completion of the incubation period the cells were vortexed gently and read on the cell analyzer using a FACStar flow cytometer (Becton-Dickinson, San Jose, CA, USA). The population of cells at G_o_/G1, S and G2/M phases were determined in control and UA plus PTX treated cells.

### 4.9. In Vivo Xenograft Animal Model

Animal experiments were carried out with the approval of the Institutional Animal Care and Use Committee (IACUC#CBNU2017-0001, 3 January 2017) of Jeonbuk National University under NIH guidelines (USA). Four-week-old female SPF/VAF immunodeficient mice were purchased from Orient Bio (Dea Jeon, Korea). After the mice had adjusted to their current conditions for two weeks, each mouse was subcutaneously inoculated with 100 µL of Matrigel containing 10^7^ human esophageal cancer cells (TE-8). After tumor implantation, the mice were randomized to four groups of five mice: (i) the untreated control group (100 μL PBS daily), (ii) the UA-treated group (UA 10 mg/kg in 100 μL of PBS daily), (iii) the PTX treated group (PTX 20 mg/kg in 100 μL of PBS twice per week), and (iv) the PTX and UA combination group (PTX 20 mg/kg in 100 μL of PBS twice per week and UA 10 mg/kg in 100 μL of PBS once daily). All drugs were administered by intraperitoneal injection. Tumor size was monitored with a caliper every three days and calculated as (width) 2 × length/2. Body weight was measured regularly before and after drug treatment every 3 days. The animals were euthanized when the tumors were 2 cm in size. Tumor tissue for H&E staining was stored in formalin, and tumor tissue for western blotting was stored at −80 °C.

### 4.10. Hematoxylin and Eosin Staining

Mouse tumor tissues were removed after euthanizing the mice, and tissues from the tumors were immediately sectioned and fixed in 10% formaldehyde at room temperature. Then the samples were embedded in paraffin, cut into 4 μm sections, and subjected to H&E staining. Histopathological analysis was performed using a light microscope.

### 4.11. Immunohistochemistry Staining

Tumor tissue specimens were fixed in 10% formalin for 2 days and embedded in paraffin to cut the sections. After deparaffinization and dehydration, the sections were incubated in anti-Ki-67 antibody (Invitrogen, Waltham, MA, USA) overnight at 4 °C. The sections were further incubated with an anti-rabbit HRP/DAB IHC kit (Abcam, Cambridge, UK) for 2 h at room temperature after washing.

### 4.12. Measurement of Serum Biochemical Levels 

Mouse blood was harvested from one-side eyeball while the mice were in deep anesthesia and then stored at −80 °C. Serum samples were extracted by centrifuge. Biochemical parameters, aspartate aminotransferase (AST, AM102-K, Asan Pharmaceutical, Seoul, Korea), alanine aminotransferase (ALT, AM103-K), blood urea nitrogen (BUN, BUN Serum Detection Kit, ARBOR ASSAYS, Ann Arbor, MI, USA), and creatinine (Creatinine Serum Detection Kit, ARBOR ASSAYS, MI, USA) were detected according to the manufacturers’ manuals.

### 4.13. Statistical Analysis

Experiments were repeated >three times. Data are expressed as means ± SE. Comparisons between groups were made using one-way ANOVA with Duncan’s multiple range test or the student’s *t*-test. A *p*-value < 0.05 or <0.01 was considered statistically significant.

## Data Availability

Not applicable.
